# Potential Add-On Effects of Manual Therapy Techniques in Migraine Patients: A Randomised Controlled Trial

**DOI:** 10.3390/jcm11164686

**Published:** 2022-08-11

**Authors:** Elena Muñoz-Gómez, Pilar Serra-Añó, Sara Mollà-Casanova, Núria Sempere-Rubio, Marta Aguilar-Rodríguez, Gemma V. Espí-López, Marta Inglés

**Affiliations:** 1 Department of Physiotherapy, Faculty of Physiotherapy, University of Valencia, Gascó Oliag Street 5, 46010 Valencia, Spain; 2 Research Unit in Clinical Biomechanics (UBIC), Department of Physiotherapy, Faculty of Physiotherapy, University of Valencia, 46010 Valencia, Spain

**Keywords:** manual therapy, articulatory techniques, soft-tissue techniques, pain, disability

## Abstract

*Objective*: To ascertain whether the combination of soft tissue and articulatory manual techniques is more effective than either one of these techniques alone for reducing migraine impact; *Methods*: Seventy-five participants with migraine were randomly divided into three groups (*n* = 25 per group): (i) soft tissue (STG), (ii) articulatory (AG), and (iii) combined treatment (STAG). Pain, frequency of occurrence, duration, disability and impact, depression and anxiety levels, and perception of change were analysed at baseline, post intervention (T2) and at four-week follow-up (T3); *Results*: STAG showed a significantly greater reduction in pain versus STG and AG at T2 (*p* < 0.001; *p* = 0.014) and at T3 (*p* < 0.001; *p* = 0.01). Furthermore, STAG achieved a significantly greater reduction in pain duration versus STG at T2 (*p* = 0.020) and T3 (*p* = 0.026) and a greater impression of change versus STG (*p* = 0.004) and AG (*p* = 0.037) at T3. Similar effects were observed in all groups for frequency of occurrence, migraine disability, impact, and depression and anxiety levels; *Conclusions*: A combined manual therapy protocol including soft tissue and articulatory techniques yields larger improvements on pain and perception of change than either technique alone, yet the three therapeutic approaches show similar benefits for reducing pain, disability and impact caused by the migraine, depression or anxiety levels.

## 1. Introduction

Migraine is a common and disabling neurological condition [1,2]. Treatments for migraine are usually focused on reducing pain, frequency of occurrence, impact and associated functional disability. Other aspects, such as emotional disorders, emotional disability, anxiety and depression have been shown to influence migraine development, prognosis, treatment and clinical outcomes [3,4,5,6,7].

Non-pharmacological treatments, such as manual therapy, non-invasive neuromodulation, or behavioural treatments may offer an alternative to drug consumption [8]. Manual therapy is the most commonly used non-pharmacological treatment for recurrent headaches [9]. The effects of manual therapy are based on the fact that the applied mechanical force may trigger neurophysiological processes at the spinal and supraspinal level on nociception modulation [10]. For example, it has been associated with hypoalgesia, afferent discharge, motoneuron pool activity and changes in muscle activity [10]. Manual therapy techniques include soft tissue and articulatory techniques, among others.

Soft tissue manual techniques intend to release myofascial structures and normalise the increased sympathetic nerve activity typically present in chronic pain [11]. Furthermore, these techniques may induce general physical relaxation, since an increase in the alpha absolute power through quantitative electroencephalography has been described [12,13]. Previous studies have suggested that soft tissue techniques may be useful in migraine patients in terms of pain and frequency of onset [14] and migraine impact [15]. Indeed, we previously reported that a protocol based on soft tissue and cranial techniques can be effective in reducing pain, frequency of onset, functional disability and use of medication in people with migraine [16].

Regarding articulatory techniques, it has been reported that they may have effects on central nervous system mechanisms responsible for pain control [17], as well as on the autonomic nervous system, reflected as changes in heart rate variability and skin conductance [18]. Articulatory techniques have shown beneficial effects on pain, frequency, disability and medication use [19,20]. We also observed that a protocol consisting of articulatory techniques could reduce pain, overall disability and medication intake, and improve the quality of life of migraine patients [20].

Some previous studies have assessed the effects of a combined protocol including soft tissue and articulatory techniques versus a no-treatment control group on pain intensity, frequency and duration of the attacks, disability and impact [21,22,23]. However, they did not assess its effectiveness on emotional factors, such as depression and anxiety, both important factors in migraine patients. Furthermore, to date, it has not been proven whether the combination of these techniques is superior to each of them implemented alone. This is interesting, firstly, because by combining the factors responsible for the positive effects of both manual therapy techniques, manual therapy effectiveness may increase in migraine patients [10]. Secondly, it is of interest because standard clinical recommends following an individualised treatment plan based on the clinical interview and physical examination [24], establishing potential triggering factors and musculoskeletal dysfunctions (i.e., joint or soft tissue disorders) that predominate in the particular patient. Intriguingly, both types of dysfunctions are usually found in people with migraine [25]. Lastly, it is important to compare the combined approach with the isolated techniques because the patient’s expectations of symptom relief have been proven to predict long-term manual therapy outcomes [26,27,28].

In view of the above, we sought to assess whether the combination of soft tissue and articulatory techniques is more effective than either one of the techniques alone on intensity, frequency of onset, duration, disability and impact, levels of depression and anxiety, and perception of change in people with migraine.

## 2. Materials and Methods

### 2.1. Participants

Participants were recruited from several primary care centres in Valencia (Spain). Inclusion criteria were: (i) aged between 18 and 50 years; (ii) diagnosis of migraine according to the ICHD-3 criteria (Headache Classification Committee of the International Headache Society [IHS]) [29]; (iii) more than four episodes per month; (iv) more than one year of evolution. Exclusion criteria were: (i) suffering from another type of primary or secondary headache; (ii) signs of involvement of the vertebral or internal carotid artery; (iii) spinal radiculopathy; (iv) vertigo; (v) decompensated blood pressure; (vi) pregnancy; (vii) undergoing pharmacological adaptation. Participants were allowed to take their usual symptomatic medication when required, but not to undergo other non-pharmacological therapies during the study.

### 2.2. Study Design

A randomized controlled trial (NCT03555214) was carried out from June to October 2018, at the University of Valencia. The sample was randomly divided into three groups: (a) soft tissue group (*n* = 25), which received a soft tissue manual therapy intervention, (b) articulatory group (AG) (*n* = 25), which received an articulatory manual therapy protocol, and (c) soft tissue and articulatory group (STAG) (*n* = 25), which received a combined protocol of (a) plus (b). The interventions consisted of one session per week for four weeks. Participants were assessed at three time points: baseline (T1), post intervention (T2), and at one-month follow-up (T3).

All volunteers were informed about the nature of the study and signed the informed consent. All procedures were carried out in accordance with the ethical standards of the Declaration of Helsinki, and the protocols were approved by the Ethics Committee of the University of Valencia (H1509655117217).

### 2.3. Randomisation, Blinding and Masking

Distribution of subjects was carried out by an investigator not involved in the evaluation or treatment of participants using the sealed envelopes method. Furthermore, participants and the investigator conducting the statistical analysis were blinded to group assignment throughout the intervention.

The effect of the interventions on variables related to pain (pain intensity, frequency of the episodes and migraine duration), migraine-associated disability and impact, emotional state (levels of depression and anxiety), and perception of change were evaluated at three time points (i.e., T1, T2 and T3).

### 2.4. Assessments

Pain intensity and frequency of episodes. They were determined based on two relevant questions of the Migraine Disability Assessment (MIDAS) that rely on pain intensity and frequency of occurrence [30]: “On a scale of 0–10 cm, how painful were these headaches on average?” and “On how many days in the last 3 months did you have a headache?”. A 1.5 cm change is considered the minimal clinically important difference (MCID) for pain intensity, which is the minimum improvement considered worthwhile by a patient or clinician [31]. For headache frequency, a one-day reduction is considered clinically meaningful [32].

Migraine duration. The duration of the migraine was assessed using a headache diary, where participants were asked to document their migraine duration, in hours.

Migraine disability. The Migraine Disability Assessment (MIDAS) is a questionnaire designed to assess disability due to migraine in relation to work, household chores, and family, social or recreational activities over a three-month period [30]. The minimal clinically important difference (MCID) has been set at a change greater than 4.5 points for disability [33]; MIDAS has good internal consistency (Cronbach’s α = 0.73–0.76) [34].

Migraine impact. The Headache Impact test (HIT-6) is a questionnaire that quantifies the impact of migraine on six domains (i.e., pain, social functioning, well-being, vitality, cognitive functioning and psychological disorders). The impact on each domain can be considered as “never”, “rarely”, “sometimes”, “very often”, or “always”. The total HIT6 score ranges from 36 to 78, where 60–78 is classified as having a very severe impact on life, 56–59 a substantial impact on life, 50–55 some impact and less than 49, little impact [35]. A decrease of 2.30 points over time reflects an improvement that can be considered clinically significant in patients with chronic headache [36]. HIT-6 is a valid and reliable tool (Cronbach’s α = 0.83–0.90) [37].

Depression. This variable was assessed by the Beck Depression Index II (BDI-II), which reports the psychological and somatic manifestations of the depressive episodes of the two preceding weeks [38]. Although the MCID for migraine patients has not been determined, the MCID for chronic pain has been set at 5 points [39]. The internal consistency of the BDI-II is Cronbach’s α = 0.90 [40].

Anxiety. The State-Trait Anxiety Inventory (STAI) is a 40-item self-reported questionnaire to measure both state and trait anxiety by means of two 20-item subscales: state-anxiety (i.e., emotional state that may vary in intensity over time) and trait-anxiety (i.e., tendency to be anxious and to perceive situations as threatening) [41]. Each item is assessed using a 4-point Likert scale, from “not at all” to “very much so” for the trait anxiety subscale, and from “almost never” to “almost always” for the state anxiety subscale. The total score ranges between 20 and 80, where 80 identifies the maximal anxiety. The MCID for migraine has not been reported, but it has been generally set at 10 points [42]. This instrument has good internal consistency (Cronbach’s α = 0.93) [41].

Self-reported perception of treatment effectiveness. It was evaluated by The Patients’ Global Impression of Change Scale (PGICS) on which the patient reflects the perception of the effectiveness of the treatment received on his or her limitation of activities, symptoms, emotions and overall quality of life [43]. The item “moderate improvement” has been reported as clinically relevant in people with chronic pain [39,44]. It has shown an excellent test-retest reliability (Intraclass Correlation Coefficient = 0.90) [45].

### 2.5. Intervention Procedures

The interventions were performed by the same physiotherapist, with more than 8 years of experience in the application of manual therapy. Prior to the intervention, all participants underwent a complete anamnesis and a pre-manipulative cervical test in order to detect and exclude any risk of cervical artery dissection [46,47]. None of the participants were excluded for this reason.

The intervention protocols performed in this study have been previously described by our research group [16,20]. Briefly, the treatment protocol applied to the STG consisted of the suboccipital inhibition technique, frontal technique, sphenoid technique, fourth ventricle technique and lumbosacral technique. The AG underwent treatment based on occiput-atlas-axis articulatory manipulation, upper cervical spine (C0–C1) mobilization, middle cervical spine (C2–C7) mobilization in supine, middle cervical spine (C2–C7) mobilization in prone, cervicothoracic junction articulatory manipulation, upper thoracic spine (T2–T6) articulatory manipulation and global sacroiliac joint articulatory manipulation. Finally, the STAG received the combination of both.

### 2.6. Statistics

Statistical analyses were performed with SPSS v.24 (IBM SPSS, Inc., Chicago, IL, USA). The effectiveness of the interventions (changes between T2 and T1, and between T3 and T1) were computed subtracting the former values from the latter values (i.e., T2–T1 and T3–T1). To compare the effectiveness of the treatments on each of the described variables, a two-way mixed multivariate analysis of variance (MANOVA) was performed, with a within-subject factor “change” having two categories (T2–T1 and T3–T1) and a between-subjects factor “group” with three categories (STG, AG, and STAG), for symptom intensity, frequency of occurrence, duration, disability and impact, and levels of depression and anxiety variables. Homoscedasticity was tested with Levene’s test, and the Bonferroni correction was used in post hoc comparisons. The between-groups and between-time analyses of the self-reported perceived change after treatment was evaluated with a Chi-square test. In addition, between-group similarity at baseline assessment was explored using one-way ANOVA (with the three groups) for the continuous variables and Chi-square test for categorical variables. Finally, in order to test whether the two conditions (i.e., episodic migraine and chronic migraine) had different responses to the interventions, an ANOVA subgroup analysis was performed. The α level was set as less than 0.05 for all tests. Effect size was calculated with Cohen’s d, small size being in the range 0.20–0.50, medium 0.50–0.80 and large > 0.80 [48]. For categorical variables, the effect size was reported by the contingency coefficient (CC).

### 2.7. Sample Size Calculation

To calculate sample size, we set a power of 80% and an effect size of *d =* 0.5, based on the results obtained for migraine disability in a previous study [14] in which a similar approach was used. Thus, a minimum sample size of twelve individuals per group was required. Considering possible dropouts, this sample size was doubled (i.e., twenty-five per group, seventy-five in total).

## 3. Results

### 3.1. Participants

**Figure 1:**
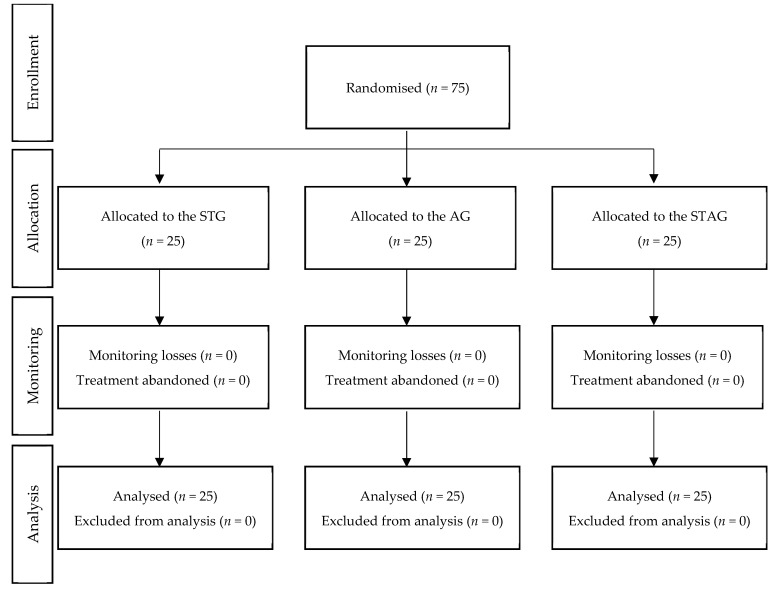
Flowchart according to CONSORT Statement for the Reporting of Randomised Trials. STG: Soft tissue group; AG: articulatory group; STAG: soft tissue and articulatory group.

As shown in Figure 1, seventy-five participants completed the study. The sample consisted of 58 (77.30%) women and 17 (22.67%) men, with a mean (SD) age of 39.01 (9.77) years. Forty-five participants (60%) were diagnosed with episodic migraine, while 30 (40%) were diagnosed with chronic migraine, and the mean (SD) time evolution was 19.97 (11.56) years. Regarding migraine characteristics, the mean (SD) pain intensity score was 7.57 (1.16), the mean (SD) frequency of the episodes was 8.47 (3.59) days per month, and the mean (SD) duration was 24.11 (20.26) hours per month. There were no significant differences between groups either in sociodemographic data or in migraine characteristics at baseline (*p* > 0.05) (Table 1). Regarding intervention-related side-effects, no serious side-effect was reported.

### 3.2. Effectiveness of the Interventions on Pain Intensity, Frequency of the Episodes and Migraine Duration

**Figure 2:**
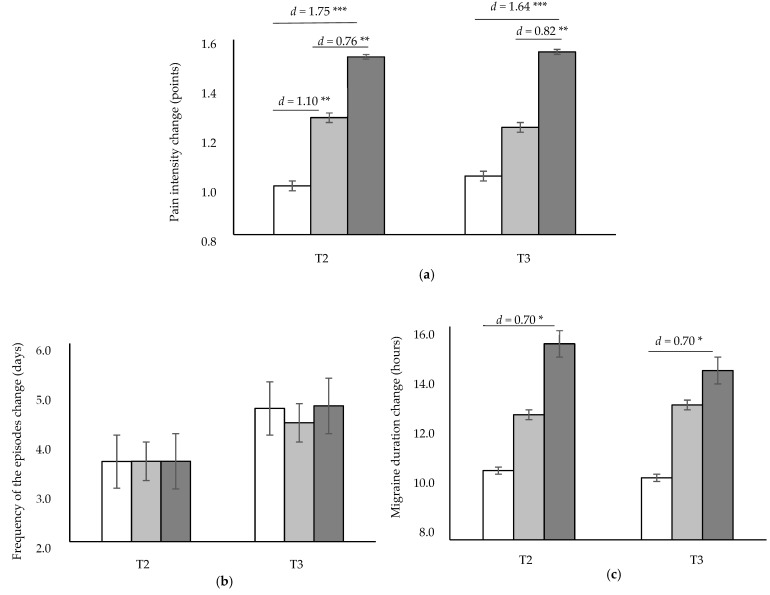
Treatment effects on changes in pain related variables: pain intensity (**a**), frequency of episodes (**b**) and migraine duration (**c**). Bars and error bars show the mean change experienced and SDs. White bars represent the soft tissue group, pale grey bars represent the articulatory group, whereas dark grey bars show the soft tissue and articulatory group. * *p* < 0.05; ** *p* ≤ 0.01; *** *p* ≤ 0.001. *d*: Cohen’s effect size (only for the significant comparisons).

The experienced improvement in pain intensity after treatment (T2) in STAG (1.46 (SD = 0.75) points) was significantly greater compared to STG (*p* < 0.001, *d* = 1.75) and AG (*p* = 0.014, *d* = 0.76). This significantly greater improvement was also observed at T3 (1.50 (SD = 0.64) points) when compared to STG (*p* < 0.001, *d* = 1.64) and AG (*p* = 0.010, *d* = 0.82). In addition, there were significant differences between AG and STG (*p* = 0.004, *d* = 1.10) at T2, with AG having a greater pain intensity improvement (Figure 2a).

When frequency of occurrence was analysed, a similar reduction was observed in all groups at T2 (*p* > 0.05) and at T3 (*p* > 0.05) (Figure 2b). Regarding migraine duration, STAG showed a greater significant reduction compared to STG both in T2 (*p* = 0.020, *d* = 0.70) and T3 (*p* = 0.026, *d* = 0.70), but reductions were similar to those achieved in the AG (Figure 2c).

### 3.3. Effectiveness of the Interventions on Migraine Disability and Impact

When analysing the effects of the three interventions on migraine disability, it was observed that this variable improved similarly in the three experimental groups at T2 and T3 (*p* > 0.05 at both time evaluations), even though the largest effect size was shown in STAG (5.68 (SD = 2.93) at T2, and 12.76 (SD = 6.97) at T3). Similarly, all three groups perceived a similar reduction in the impact of migraine at T2 and T3 (*p* > 0.05), although the largest change was perceived by STAG, with a reduction of 9.68 (SD = 4.83) points at T2, and 8.80 (SD = 5.39) points at T3. Table 2 summarises the changes observed for both variables (i.e., migraine disability and impact) at T2 and T3, when compared to baseline.

### 3.4. Effectiveness of the Interventions on Depression and Anxiety Levels

Reductions obtained in depression levels were similar between groups at T2 and T3 (*p* > 0.05). Similarly, reductions in anxiety levels, either total, or on both subscales (state and trait), were similar in all three groups at T2 and T3 (*p* > 0.05) (Table 2).

### 3.5. Effectiveness of Interventions on Patient Global Impression of Change

Regarding the self-reported perception of change, 80% of STAG participants reported “Much improved” and “Very much improved”, while only 52% of STG and 52% of AG participants reported such statements at T2; however, there were no significant differences between groups at that time assessment (*p* > 0.05). At T3, 48% of STAG participants reported “Much improved”, whereas only 12% of STG and 20% of AG participants perceived that change. There were significant differences between STG and STAG (χ^2^ = 11.00, *p* = 0.004, CC = 0.43), and between AG and STAG (χ^2^ = 6.61, *p* = 0.037, CC = 0.34) at T3 (Table 3).

### 3.6. Subgroup Analysis by Diagnosis

There was no significant interaction between the two conditions (i.e., episodic migraine and chronic migraine) and the studied interventions (STG, AG and STAG) in the variables pain intensity, frequency of occurrence, duration, disability, and impact, and in the levels of depression and anxiety, either after the intervention (*p* > 0.05) or at follow-up evaluation (*p* > 0.05).

## 4. Discussion

The results of the current study show that all three intervention protocols were effective in reducing pain, disability and impact in people with migraine, yet no significant improvements were observed in depression and anxiety levels. It is noteworthy that the effects on pain and perception of change were largest when combining soft tissue and articulatory techniques. To our knowledge, this is the first study to compare the effectiveness of a combined protocol including soft tissue and articulatory techniques and that of the two techniques alone in people with migraine.

Episodic and chronic pain interferes with the patient’s life, affecting his or her physical and emotional state, as well as the recovery process [49,50]. Moreover, recurrent episodes contribute to migraine chronification [5,6]. Our results show that the combined group achieved better improvements than either of the groups receiving isolated techniques on pain intensity, with a large significant difference after the intervention (*d* = 1.58) and at the 4-week follow-up (*d* = 1.45). Additionally, the STAG was the only group that achieved the MCID (1.5 points) at T2 and T3. It has been suggested that manual therapy techniques initiate a cascade of neurophysiological responses from the peripheral nervous system (modulation of inflammatory response), and the central nervous system at spinal (activation of somato-autonomic reflexes) and supraspinal levels (regulation of brain areas such as the anterior cingulate cortex, amygdala or periaqueductal grey), which may be responsible for pain reduction [10,51]. In this regard, although the different biomechanical and neurophysiological effects specific to types of MT are not well established, our results may suggest a potential cumulation of the physiological effects involved in both MT techniques.

Regarding frequency of occurrence, the combined protocol was equally effective as the isolated-technique formats, and hence, the three of them achieved the MCID. In line with these results, previous studies with a similar frequency of treatment to that reported in our study (i.e., one session per week maximum) have reported a reduction in frequency of occurrence after applying manual therapy based on soft tissue techniques (reduction of 1.96 days) [52], articulatory techniques (reduction of 2.6 days) [19] or a combination of both (ranging from 2.92 to 4.49) [21,22,23] in people with migraine. Bevilaqua et al. [21] reported a greater reduction in the frequency of the episodes than that obtained in the current study (4.49 vs. 2.84 days) after applying cervical mobilisations and massage and myofascial release twice a week over a 4-week period, and thus, a higher frequency of treatment may have yielded better results.

When the effect on duration of the episodes was analysed, the number of hours per episode for STAG reduced significantly more than for STG (i.e., 14.71 vs. 5.20), with a large effect size at T2 (*d* = 1.36) and at T3 (*d* = 1.20). It is possible that the added effect of joint mobilisations and manipulations could justify such results. In this regard, evidence suggests that spinal mobilisations cause neurophysiological effects, resulting in hypoalgesia (local and/or distal to the mobilisation site), sympathoexcitation, and improved muscle function [53], which, in addition to the effects of soft tissue techniques, may have enhanced the effects. Although no previous study has compared the three therapeutic approaches, other authors have also observed a beneficial effect on this variable after applying a combined manual therapy protocol [19,52].

Recently, migraine disability as assessed by MIDAS, migraine impact, as assessed by HIT-6 and frequency of episodes have been recommended as the most useful outcome measures for research on the effectiveness of non-pharmacological interventions for migraine [54]. In this regard, when the MIDAS total score was analysed, we observed that all three groups achieved the MCID at T2 and T3, except for the AG at T2. Furthermore, the STAG achieved a larger effect size (*d =* 0.90) when compared to STG (*d* = 0.67) and AG (*d* = 0.60). Regarding migraine impact, our results showed similar changes with the three interventions; however, the largest effect size was again achieved by the STAG, compared to the STG and AG after the intervention (*d =* 1.84 vs. *d =* 1.23 vs. *d =* 1.23, respectively). The largest effect size on migraine disability and impact in the STAG may be related to the larger decrease in pain, since the longer duration and more frequent migraine attacks are predictors of both disability and detrimental effects on quality of life in migraineurs [55]. In line with our results, Voigt et al. [23] reported an improvement in disability after applying a combined manual therapy intervention (5 sessions over a 10-week period), yet they did not describe the full protocol (thus preventing replication) nor did they study the effects of the techniques separately. Furthermore, some studies have shown that soft tissue techniques alone achieve smaller improvements in disability that in some cases failed to reach the MCID, with a range from approximately 4 to 5.67 points [15,56,57]. On the other hand, Tuchin et al. [58] observed an improvement in disability after applying a protocol with articulatory techniques alone in people with migraine, but the improvement (i.e., 6.8 points) was reported in a disability diary, and not with a validated questionnaire. These results suggest that the addition of articulatory techniques could be responsible for the greatest reduction in disability, as has been recently supported [59]. In contrast with our results, Gandolfi et al. [22] reported that a combined protocol including myofascial release and manipulative articulatory techniques did not improve MIDAS and HIT-6 total scores, when compared to a control group receiving transcutaneous electrical nerve stimulation (TENS) in the upper trapezius. This could be explained in that the effects were not assessed immediately after the 4-week intervention, but after 8 weeks. In addition, participants simultaneously underwent onabotulinumtoxin A injection, due to chronic intractable migraine, so the results are not entirely comparable with ours.

Regarding emotional status variables, we found that manual therapy had no effect on either depression or anxiety levels, and thus, no difference between groups was found. Our results cannot be compared to other studies, since no previous study has analysed or compared the effect of manual therapy on these variables in migraine patients. However, it is important to study mood disorders in migraine patients, since they are relevant psychological comorbidities, and they have a negative impact on prevalence, prognosis, treatment and clinical outcomes of migraine [5,6]. Previous studies on tension-type headache patients have shown that a manual therapy protocol combining soft tissue and articulatory techniques improved depression and anxiety levels [60]. The fact that the sample included in our study had low basal depression and anxiety levels may be the reason why we did not observe positive effects on these variables.

Finally, the patient’s self-perception of change is considered an important factor in the management of patients, because it is a composite assessment of treatment effects on headache, associated symptoms, and tolerability [61]. Our results showed that 80% of STAG participants achieved the item “moderately improved”, which has been reported as clinically relevant in people with chronic pain [39,44]. This is of particular interest, because the clinically relevant changes in migraine frequency and intensity have been associated with a better perception of change after a manual therapy protocol in migraine patients [21]. Furthermore, STAG perceived significantly greater improvements than STG at T3, which may indicate that the improvements achieved by this group are maintained longer in time.

Overall, we are able to report that all three studied intervention approaches exert a beneficial effect on pain, disability, impact and perception of change, but not on depression and anxiety levels in people with migraine. However, the combined approach showed greater improvements in pain than either of the isolated manual techniques, and larger benefits on pain duration and perception of change than soft tissue techniques. Thus, different treatment approaches might be used depending on the patient’s major symptoms or characteristics. Furthermore, the findings of this study can be easily applied in common clinical practice, since they involve easy-to-implement, inexpensive, well-tolerated and safe interventions [62].

### Limitations

This study has some limitations. Firstly, we recruited mostly women from a primary care setting and from one particular city. However, it was difficult to match the number of men and women, since migraine is twice as prevalent in women. Another limitation is that the combined group received an intervention that lasted twice as long as that of the other groups separately. Furthermore, longer follow-up periods would be desirable to confirm the duration of the beneficial effects. Finally, in order to prevent medication overuse headaches, future studies should keep a record of the use of drugs during the application of manual therapy techniques.

## 5. Conclusions

Without disregarding the aforementioned limitations, a combined manual therapy protocol including soft tissue and articulatory techniques yields larger improvements on pain intensity and perception of change than either of the isolated techniques, yet the three manual therapy protocols achieve similar benefits for reducing pain, disability and impact caused by the migraine. Nevertheless, none of the three approaches improve depression or anxiety levels.

## Figures and Tables

**Table 1:** Descriptive analysis of the sample at baseline.

	STG *n* = 25	AG *n* = 25	STAG *n* = 25	Total *n* = 75
Medication				
Preventive	2 (8%)	8 (32%)	9 (36%)	19 (25.33%)
Symptomatic	23 (92%)	16 (64%)	16 (64%)	55 (73.33%)
None	0 (0%)	1 (4%)	0 (0%)	1 (1.33%)
Migraine duration (hours)	24.24 (24.62)	23.20 (20.28)	24.88 (15.73)	24.11 (20.26)
Pain intensity (MIDAS)	7.28 (1.09)	7.40 (1.13)	7.56 (1.10)	7.41 (1.10)
Frequency of occurrence (MIDAS)	25.08 (11.02)	24.84 (12.26)	24.48 (11.41)	24.8 (11.42)
Migraine Disability (MIDAS)	35.76 (21.06)	34.64 (20.23)	35.72 (15.84)	35.37 (18.93)
Migraine Impact (HIT-6)	63.44 (4.34)	64.44 (6.49)	64.00 (4.7)	63.96 (5.21)
Depression (BDI-II)	7.68 (6.96)	8.64 (7.92)	8.00 (5.21)	8.11 (6.71)
State-Anxiety (STAI-state)	40.84 (13.38)	43.76 (13.94)	43.76 (12.32)	42.79 (13.12)
Trait-Anxiety (STAI-trait)	38.64 (12.46)	40.32 (12.85)	40.00 (12.57)	39.65 (12.48)
Anxiety (STAI total)	39.74 (12.44)	42.04 (13.13)	41.88 (11.85)	41.22 (12.36)

Data is shown as absolute frequency (percentage) for the categorical variables and mean (standard deviation) for the continuous variables. STG = soft tissue group; AG = articulatory group; STAG = soft tissue and articulatory group.

**Table 2:** Comparison of the changes observed between the three intervention groups in migraine disability and impact, and levels of depression and anxiety.

	Change T2–T1						Change T3–T1					
	STG	AG	STAG	STG vs. STAG	AG vs. STAG	STG vs. AG	STG	AG	STAG	STG vs. STAG	AG vs. STAG	STG vs. AG
Migraine Disability (MIDAS)	6.00 (3.28)	4.48 (2.71)	5.68 (2.93)	*d =* −0.10	*d =* 0.43	*d =* −0.51	12.00 (8.58)	10.36 (7.11)	12.76 (6.97)	*d =* 0.10	*d =* 0.34	*d =* −0.21
Migraine Impact (HIT-6)	7.75 (7.96)	8.48 (4.02)	9.68 (4.83)	*d =* 0.30	*d =* 0.27	*d =* 0.12	6.71 (5.83)	7.56 (4.44)	8.80 (5.39)	*d =* 0.37	*d =* 0.25	*d =* 0.17
Depression (BDI-II)	2.38 (9.61)	1.88 (3.60)	2.52 (3.51)	*d =* 0.02	*d =* 0.18	*d =* −0.08	1.75 (4.67)	0.83 (4.64)	1.52 (4.00)	*d =* −0.05	*d =* 0.16	*d =* −0.20
State-Anxiety (STAI-state)	5.23 (16.44)	2.25 (10.33)	5.36 (5.50)	*d =* 0.01	*d =* 0.39	*d =* −0.22	2.77 (7.98)	3.67 (9.36)	5.16 (5.91)	*d =* 0.34	*d =* 0.20	*d =* 0.10
Trait-Anxiety (STAI-trait)	5.19 (12.13)	4.16 (5.19)	4.40 (8.71)	*d =* −0.08	*d =* 0.03	*d =* −0.12	5.19 (6.47)	4.56 (6.25)	4.16 (8.48)	*d =* −0.14	*d =* −0.05	*d =* −0.10
Anxiety (STAI total)	4.13 (16.09)	3.06 (6.90)	4.88 (6.41)	*d =* 0.07	*d =* 0.27	*d =* −0.09	3.87 (5.67)	3.77 (5.98)	4.66 (6.68)	*d =* 0.13	*d =* 0.14	*d =* −0.02

Data shown as mean (SD). STG = soft tissue group; AG = articulatory group; STAG = soft tissue and articulatory group. T1 = baseline; T2 = post intervention; T3 = 4-week follow-up. MIDAS = Migraine Disability Assessment; HIT-6 = Headache Impact test; BDI-II = Beck Depression Index II; STAI = State-Trait Anxiety Index. Effect size between groups shown as Cohen’s.

**Table 3:** Between-group comparisons in post-intervention and follow-up assessments of patient global impression of change.

	T2			T3		
	STG	AG	STAG	STG	AG	STAG
Patient global impression of change (PGICS)						
No change	7 (28)	3 (12)	1 (4)	8 (32)	6 (24)	1 (4)
Small improvement	5 (20)	9 (36)	4 (16)	14 (56)	14 (56)	12 (48)
Moderate improvement	11 (44)	12 (48)	18 (72)	3 (12) **	5 (20) *	12 (48)
Large improvement	2 (8)	1 (4)	2 (8)	0 (0)	0 (0)	0 (0)

Data shown as absolute frequency (percentage). STG = soft tissue group; AG = articulatory group; STAG = soft tissue and articulatory group. T2 = post intervention; T3 = 4-week follow-up. * *p* < 0.05 vs. STAG; ** *p* < 0.01.

## Data Availability

Data analyzed in the current study are available from the corresponding author upon reasonable request.
